# Development of an implantable collamer lens sizing model: a retrospective study using ANTERION swept-source optical coherence tomography and a literature review

**DOI:** 10.1186/s12886-023-02814-7

**Published:** 2023-02-10

**Authors:** Taein Kim, Su Jeong Kim, Bo Young Lee, Hye Jin Cho, Beom Gi Sa, Ik Hee Ryu, Jin Kuk Kim, In Sik Lee, Eoksoo Han, Hyungsu Kim, Tae Keun Yoo

**Affiliations:** 1 VISUWORKS, Seoul, South Korea; 2 Department of Refractive Surgery, B&VIIT Eye Center, 1317-23 Seocho-Dong, Seocho-Gu, Seoul, South Korea; 3 Electronics and Telecommunications Research Institute (ETRI), Daejeon, South Korea; 4 Yonsei Eye Clinic, Seoul, South Korea

**Keywords:** ANTERION, Implantable collamer lens, Lens sizing, Postoperative vault

## Abstract

**Background:**

Optimal sizing for phakic intraocular lens (EVO-ICL with KS-AquaPort) implantation plays an important role in preventing postoperative complications. We aimed to formulate optimal lens sizing using ocular biometric parameters measured with a Heidelberg anterior segment optical coherence tomography (AS-OCT) device.

**Methods:**

We retrospectively analyzed 892 eyes of 471 healthy subjects treated with an intraocular collamer lens (ICL) and assigned them to either the development (80%) or validation (20%) set. We built vault prediction models using the development set via classic linear regression methods as well as partial least squares and least absolute shrinkage and selection operator (LASSO) regression techniques. We evaluated prediction abilities based on the Bayesian information criterion (BIC) to select the best prediction model. The performance was measured using Pearson’s correlation coefficient and the mean squared error (MAE) between the achieved and predicted results.

**Results:**

Measurements of aqueous depth (AQD), anterior chamber volume, anterior chamber angle (ACA) distance, spur-to-spur distance, crystalline lens thickness (LT), and white-to-white distance from ANTERION were highly associated with the ICL vault. The LASSO model using the AQD, ACA distance, and LT showed the best BIC results for postoperative ICL vault prediction. In the validation dataset, the LASSO model showed the strongest correlation (r = 0.582, P < 0.001) and the lowest MAE (104.7 μm).

**Conclusion:**

This is the first study to develop a postoperative ICL vault prediction and lens-sizing model based on the ANTERION. As the measurements from ANTERION and other AS-OCT devices are not interchangeable, ANTERION may be used for optimal ICL sizing using our formula. Because our model was developed based on the East Asian population, further studies are needed to explore the role of this prediction model in different populations.

**Supplementary Information:**

The online version contains supplementary material available at 10.1186/s12886-023-02814-7.

## Background

The first phakic intraocular lens (IOL) is implanted in the anterior chamber angle (ACA), which causes endothelial decompensation and glaucoma [1]. Iris-claw (iris-fixated) IOLs located in the anterior chamber without endothelial touching have been used to correct refractive errors, but significant endothelial cell loss in some patients has been reported [2]. Posterior chamber phakic IOLs, which are located behind the iris, have been introduced most recently to minimize the effect of IOL on corneal and anterior chamber angles. The implantable collamer lens (EVO Visian implantable collamer lens [ICL] with KS-AquaPort, STAAR Surgical, USA) has been widely used for phakic intraocular lens implantation [3]. This surgical procedure is currently acknowledged as a safe and effective method for vision correction for a wide range of refractive errors. It almost preserves the cornea and accommodation function of the crystalline lens after surgery. Therefore, ICL is an important surgical option for high levels of myopia, hyperopia, and astigmatism, which cannot be corrected by corneal laser ablation. In this surgical procedure, an ICL is implanted in the ciliary sulcus in the posterior chamber, far from the corneal endothelium [4]. Thus, irreversible damage to the corneal endothelium caused by IOL may be minimized, and IOL implantation is reversible and replaceable with another IOL.

The postoperative vault, which is the distance between the IOL and crystalline lens, is an important factor in selecting the optimal IOL size to reduce complications [5]. The consensus is that the ideal vault, which is the gap between the IOL and crystalline lens, should be approximately 500 μm to prevent postoperative complications. A very low vault may be associated with subcapsular cataract [6]. In addition, a vault that is too high significantly reduces the ACA opening. Too large IOL may cause iridocorneal touch and ACA block [7]. Consequently, it may increase the intraocular pressure (IOP) and reduce the number of corneal endothelial cells [8]. The abnormal position of the ICL is also associated with an improper high vault [9]. Because the postoperative vault depends on the anatomy of the anterior chamber, space between the iris and crystalline lens, and size of the ICL, it is essential to select an appropriate ICL size before surgery. The postoperative vault is often much higher or lower than intended, and reoperation is sometimes needed to avoid vision-related complications [10].

**Fig. 1:**
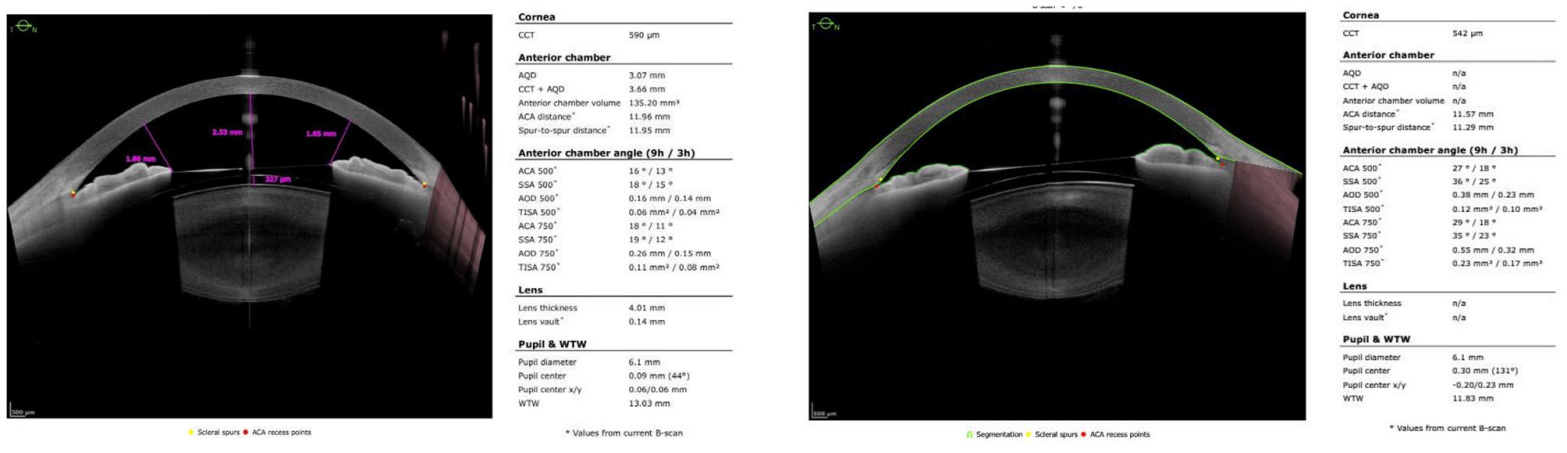
Examples of the ANTERION result pages for anterior segment measurements

Although the manufacturer provides ICL sizing based on corneal size and anterior chamber depth (ACD), directly measuring the anatomical space into which the ICL is located is more accurate in determining the optimal ICL size [11]. Anterior segment optical coherence tomography (AS-OCT) was recently developed to measure the anatomy of the anterior segment of the eye more accurately. CASIA2 (Tomey, Nagoya, Japan) and ANTERION (Heidelberg Engineering GmbH, Heidelberg, Germany) are swept-source AS-OCT devices that provide rapid image acquisition and adequate visualization depth. ANTERION can perform a wider and deeper scan than other AS-OCT devices [12]. It uses a 1300 nm infrared light source to capture the anterior segments with a high resolution (< 10 μm). The embedded software measures the anterior chamber dimensions in six evenly spaced radial scans over 12 angle locations. The infrared camera captures horizontal cross-sectional images measuring the entire anterior segment and laterals and performs en face imaging of the subject’s eye (Fig. 1). Furthermore, previous studies have reported that it outperforms CASIA2 and IOLMaster in terms of intra-device repeatability [13],[14]. Therefore, the use of ANTERION for clinical purposes is on the rise. However, CASIA2 has been widely used for ICL implantation surgery with several lens-sizing formulas [15], whereas no studies have been conducted on lens sizing using biometry from ANTERION.

Previous studies have shown that the structures of the posterior chamber, especially the ciliary sulcus, measured by ultrasound biomicroscopy (UBM) or ultrasound scanners, might provide better vault predictability [16]. UBM has the advantage of visualizing the structures behind the iris, which cannot be observed in AS-OCT images [17]. However, measurement using UBM is uncomfortable because of the placement of an eyecup between the lids and is very time-consuming in the clinic [4]. These difficulties may lead to a low repeatability. Therefore, AS-OCT measurement, which has a higher scanning speed and better resolution of the anterior chamber, has been the gold standard method for capturing the structures of the anterior segment for ICL surgery in many clinics.

According to a recent study, biometry measurements from ANTERION and CASIA2 are not interchangeable [13]. Therefore, a new ICL sizing formula is needed for clinics that use ANTERION to achieve more accurate ICL sizing and postoperative vault prediction. In this study, we developed an ICL sizing formula for the ANTERION AS-OCT device. We evaluated the effect of biometry measurements from ANTERION and performed regression analysis to calculate the optimal ICL size on the achieved vault.

## Materials and methods

### Dataset

We retrospectively collected the preoperative and postoperative ocular measurement data from the B&VIIT Eye Center (Seoul, South Korea). This study was approved by the institutional review board of the Korean National Institute for Bioethics Policy (No. 2021-3387-001). The patients underwent refractive surgery with posterior phakic intraocular lens implantation using ICL (V4c and V5 models, EVO Visian ICL with KS-AquaPort) from February 2021 to September 2021. The inclusion criteria for this study were as follows: age between 18 and 50 years, stable refraction, − 0.50 to − 20.00 diopters of hyperopia or myopia with astigmatism of 5.50 D or less, and availability of the preoperative scanning results of ANTERION, CASIA2, and the ICL vault at 1 month post-surgery. AS-OCT was performed under dark-light conditions using blackout curtains. To ensure a non-accommodative state, the patient was asked to stay far away during the examination. One trained observer marked the scleral spurs in each image during the examination. We also measured the anterior chamber width (ACW) and crystalline lens rise (CLR) using CASIA2 to compare the developed formula with the NK formula. It should be noted that CLR is currently not measured using ANTERION (Fig. 1).

**Fig. 2:**
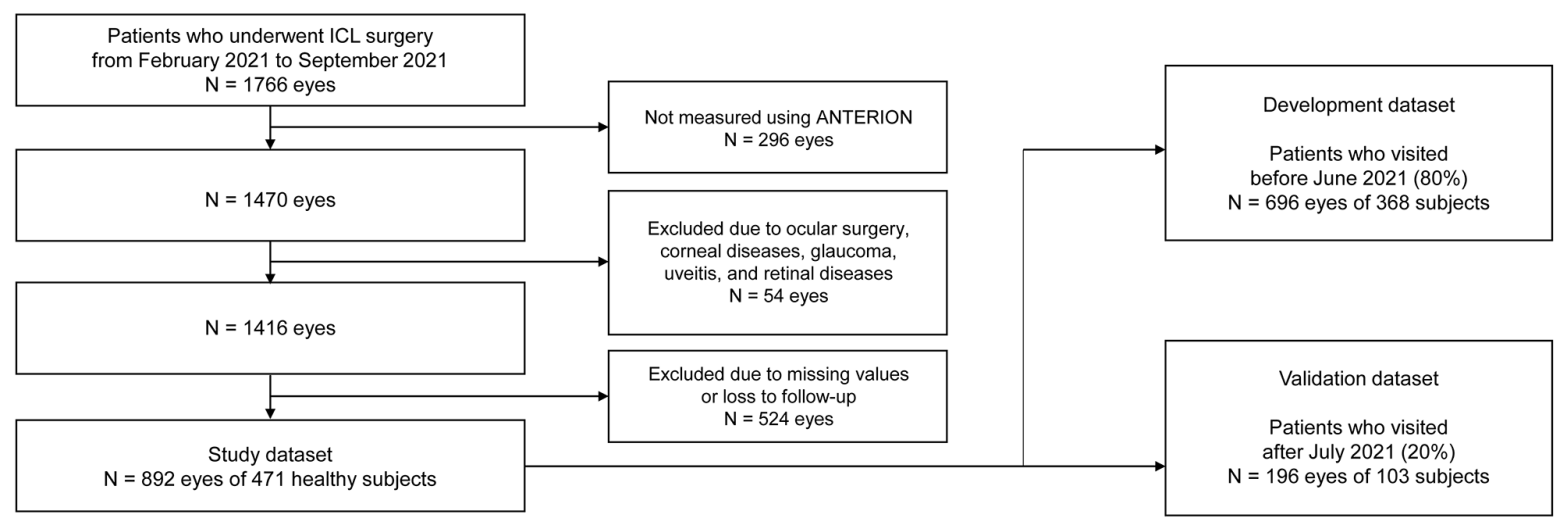
Workflow for data management for the development of regression models using ANTERION for ICL sizing

The workflow for data management is shown in Fig. 2. Subjects with a history of ocular surgery, corneal disease, glaucoma, uveitis, or retinal disease were excluded from the study. Patients with missing data were excluded from this study. This study included 892 eyes of 471 healthy subjects treated with ICLs. To design the retrospective development of the model and prospective validation via chronological splitting, we assigned patients who visited before June 2021 (80%, n = 696 eyes of 368 subjects) to the development dataset and those who visited after July 2021 (20%, n = 196 eyes of 103 subjects) to the test validation dataset.

This study aimed to develop a formula for postoperative vault prediction to select the optimal ICL size. Because there are currently four commercial ICL sizes (12.1, 12.6, 13.2, and 13.7 mm), surgeons should select the one that achieves the best outcome. The ICL vault prediction formulas were developed using the development dataset via various linear regression techniques, including ICL size and other measurements from ANTERION. Postoperative vault was measured using ANTERION. We set a postoperative ICL vault of 500 μm as the best result for obtaining the optimal ICL size. Therefore, in the validation stage, we selected the ICL size that yielded the predicted result of the ICL vault closest to 500 μm.

Phakic ICL implantation surgeries were performed using standard methods as described in previous studies [5],[18]. The lens was implanted in the posterior chamber via a 3.0-mm-sized temporal clear corneal incision. Expert surgeons (IHR, JKK, and ISL) with an average experience of 10 years determined the size of the ICL by considering the manufacturer’s nomogram (based on ACD and white-to-white distance [WTW]), an in-house developed nomogram [5], and ocular measurements of the ACA and spur-to-spur (STS) distances from ANTERION. The in-house nomogram mainly used ACW, ACD, ICL power, CLR, angle-to-angle distance (ATA), pupil size, and WTW to calculate the postoperative vaults. Before surgery, the surgeons determined the lens size for each patient based on postoperative IOL vault predictions, with a target size of 500 μm. All experts were board-certified ophthalmologists with an average experience of 5 years in ICL surgery.

### Regression techniques

We constructed six ICL vault prediction models: STAAR nomogram-based, KS formula-based, forward stepwise selection, backward stepwise selection, partial least squares (PLS), and least absolute shrinkage and selection operator (LASSO) regression models. Multivariable linear regression models (STAAR nomogram-based, KS formula-based, forward stepwise selection, and backward stepwise selection models) were fitted using the least-squares approach in a standard manner, provided by the statistical software SPSS Statistics v.24 (IBM Corp., Armonk, NY, USA). PLS and LASSO are well-known advanced regression methods for dimension reduction and robust prediction in biomedical fields [19]. These techniques are fundamentally based on familiar expressions used for multivariate linear regression. The PLS technique analyzes new vector components by combining predictors. A previous study reported that it performed well in ICL sizing using Visante OCT (Carl Zeiss, Germany) [20]. LASSO leads to a sparse regression solution for the coefficients corresponding to the most important predictors [21]. LASSO shows better identification of predictors than other regression methods [22] and outperforms machine learning techniques in predicting clinical outcomes in ophthalmology [23]. We used SPSS 23.0 (SPSS Inc., Chicago, USA) for classic statistical analysis, as well as MATLAB R2021a (MathWorks Inc., Natick, MA, USA) for PLS (“plsregress” function) and LASSO (“lasso” function) techniques.

The input variables from preoperative ANTERION measurements included central corneal thickness (CCT), aqueous depth (AQD), anterior chamber volume, ACA distance, STS distance, lens thickness (LT), pupil diameter, and WTW. We also analyzed age, sex, spherical equivalent, mean keratometry (K), ICL power, ICL type (toric or non-toric lens), and ICL size to build the ICL vault prediction model.

### Model selection

After the training process with the entire development dataset, we evaluated the prediction abilities based on the Bayesian information criterion (BIC) to select the best prediction model. BIC is a reliable indicator for comparing the effectiveness of prediction models and has been widely used in prediction model selection when different numbers of input variables are included [22]. The BIC penalizes the number of variables to avoid an unstable or overfitting regression model. In this study, BIC is expressed as follows [24]:

$$BIC=k \text{log}\left(n\right)-2\text{log}\left(\widehat{L}\right)= k \text{log}\left(n\right)+n\text{log}\left(\frac{RSS}{n}\right)$$

where $k$ is the number of predictors, $n$ is the number of samples in the validation set, $\widehat{L}$ is the likelihood of the model, and $RSS$ is the residual sum of squares of the regression result. The best model yielded the lowest BIC value and was the most effective model without overfitting. In this study, we determined the best model based on the BIC values in the validation.

We also compared our method with the NK formula based on ACW and CLR. Since the participants in this study had a different distribution of CLR than those in the original study (CLR value in our development set from Table 1 = − 0.077 ± 0.138 mm; CLR value in the Japanese population = 0.039 ± 0.183 mm), we applied the coefficient values of the NK formula optimized to the Korean population. Modified NK formula developed for the previous study [5] is expressed as follows:

$$\begin{array}{l}{\rm{Optimal\ ICL\ size (mm) = 8}}{\rm{.16 + 0}}{\rm{.36}} \times \\{\rm{ACW(mm) + 1}}{\rm{.03}} \times {\rm{CLR (mm)}}\end{array}$$

Pearson’s correlation coefficients and mean squared error (MAE) between the achieved and predicted vault values were used to evaluate the regression models. All statistical tests were performed in a two-sided manner, with the significance level set at a P-value < 0.050.

**Table 1:** Preoperative demographics and postoperative ICL vaults of the study participants

	Development set	Validation set	P-value
Number of eyes (patients)	696 (368)	196 (103)	
Age (years)	25.97 ± 5.46	26.61 ± 5.50	0.147
Sex, female (%)	409 (58.8)	119 (60.7)	0.681
Spherical equivalent, SE (D)	-8.59 ± 2.32	-8.56 ± 2.10	0.872
Mean K (D)	43.87 ± 1.40	43.80 ± 1.42	0.542
ICL power (D)	-10.48 ± 2.46	-10.45 ± 2.12	0.878
Toric lens (%)	389 (55.9)	111 (56.6)	0.871
Achieved ICL size			0.437
12.1 mm (%)	289 (41.5)	73 (37.2)	
12.6 mm (%)	355 (51.0)	112 (57.1)	
13.2 mm (%)	51 (7.3)	11 (5.6)	
13.7 mm (%)	1 (0.1)	0 (0.0)	
ANTERION OCT parameters			
Central corneal thickness, CCT (µm)	531.08 ± 34.70	525.49 ± 39.28	0.072
Aqueous depth, AQD (mm)	3.31 ± 0.25	3.33 ± 0.22	0.143
Anterior chamber volume (µL)	194.61 ± 29.44	197.38 ± 28.03	0.229
ACA distance (mm)	11.82 ± 0.38	11.88 ± 0.39	0.044
STS distance (mm)	11.67 ± 0.40	11.74 ± 0.39	0.006
Lens thickness, LT (mm)	3.68 ± 0.22	3.70 ± 0.23	0.343
Pupil diameter (mm)	5.72 ± 1.12	5.61 ± 1.21	0.235
White-to-white distance, WTW (mm)	11.99 ± 0.41	12.01 ± 0.39	0.596
CASIA2 OCT parameters of NK formula			
Anterior chamber width, ACW (mm)	11.77 ± 0.40	11.84 ± 0.43	0.058
Crystalline lens rise, CLR (µm)	-76.91 ± 173.79	-73.43 ± 182.86	0.845
Postoperative achieved ICL vault (µm)	513.94 ± 162.76	514.01 ± 148.06	0.996

Data are presented as the mean ± standard deviation unless noted otherwise

*ACA* anterior chamber angle, *ICL* implantable collamer lens, *OCT* optical coherence tomography, *STS* spur-to-spur

## Results

The demographics and measurements of the study participants in the development and validation datasets are shown in Table 1. Except for ACA distance (P = 0.044) and STS distance (P = 0.006), there were no significant differences between the development and validation datasets. There was only one case using an ICL with a size of 13.7 mm in the development dataset and none in the validation set. The mean achieved ICL vault values at 1 month post-surgery were 513.94 ± 162.76 μm in the development dataset and 514.01 ± 148.06 μm in the validation dataset. According to the retrospective chart review, all surgeries were uneventful and there were no vision-threatening complications.

Table 2 shows the descriptive statistics of the preoperative variables and ICL size to predict postoperative ICL vaults. The coefficients of the descriptive statistics were calculated using correlation coefficients and standard multivariable regression. The predictors, including AQD (r = 0.441, P < 0.001), LT (r = − 0.418, P < 0.001), and ICL size (r = 0.392, P < 0.001), showed a very strong correlation with the postoperative vault. The anterior chamber volume (ACV), ACA distance, STS distance, and WTW were also significantly correlated. In the multivariable regression model using all predictors, ICL size, AQD, LT, and ACA distance had an impact of more than 10% on the ICL vault calculation, with statistical significance.

**Table 2:** Correlation analysis and multivariable linear regression results for postoperative vault prediction using ANTERION

	Correlation with postoperative vault		Multivariable linear regression for postoperative vault prediction		
	Pearson’s correlation coefficient	P-value	Unstandardized coefficient	Impact on vault calculation (%)	P-value
Age (years)	-0.216	< 0.001	2.74	2.9	0.011
Sex (male: 0, female: 1)	-0.042	0.267	38.94	3.7	< 0.001
Spherical equivalent, SE (D)	-0.096	0.011	-9.78	4.4	0.420
Mean K (D)	-0.094	0.013	-32.73	8.9	< 0.001
ICL power (D)	-0.151	< 0.001	-6.00	2.9	0.615
Toric lens (non-toric: 0, toric: 1)	-0.172	< 0.001	-37.89	3.7	0.018
Achieved ICL size (mm)	0.392	< 0.001	298.02	18.9	< 0.001
Central corneal thickness, CCT (µm)	-0.049	0.197	-0.33	2.3	0.026
Aqueous depth, AQD (mm)	0.441	< 0.001	308.20	15.0	< 0.001
Anterior chamber volume, ACV (µL)	0.380	< 0.001	-1.43	8.2	0.010
ACA distance (mm)	0.218	< 0.001	-134.83	10.1	< 0.001
STS distance (mm)	0.214	< 0.001	2.44	0.2	0.934
Lens thickness, LT (mm)	-0.418	< 0.001	-271.83	11.4	< 0.001
Pupil diameter (mm)	0.159	< 0.001	15.66	3.4	0.004
White-to-white distance, WTW (mm)	0.215	< 0.001	-50.90	4.0	0.012

*ICL* intraocular collamer lens

Table 3 presents the prediction results of the regression models developed using the development and validation datasets. In the self-validation of the development dataset, PLS showed the best prediction performance using age, sex, toric lens, mean K, ICL size, ICL power, CCT, AQD, ACV, LT, ACA distance, pupil diameter, and WTW (13 predictors) as well as the strongest correlation (r = 0.664, P < 0.001) and lowest MAE (96.9 μm). When we investigated the BIC to consider the effectiveness of the vault prediction models in the development dataset, the LASSO model using ICL size, AQD, ACA distance, and LT yielded the best results. The final LASSO model used fewer predictors, with a smaller loss in prediction than the PLS model. In the validation dataset, the LASSO model showed the strongest correlation (r = 0.582, P < 0.001) and the lowest MAE (104.7 μm). It also had the best effectiveness for vault prediction according to BIC among the regression models.

**Table 3:** Multiple regression analysis for postoperative ICL vault prediction in the development dataset

	Combination of variables	Pearson’s correlation coefficient	P-value for correlation	MAE (µm)	Bayesian information criterion
Development set (self-validation) (N = 696)	Selection from STAAR nomogram - ICL size, WTW, ACD	0.516	< 0.001	109.8	6891.4
	Selection from KS formula – ICL size, ATA (= ACA distance)	0.427	< 0.001	118.3	6961.0
	Forward stepwise selection – age, mean K, ICL size, ICL power, CCT, AQD, ACA distance, LT	0.636	< 0.001	101.7	6789.2
	Backward stepwise selection – age, toric lens, SE, mean K, ICL size, AQD, ACA distance, LT, WTW	0.608	< 0.001	98.3	6810.1
	Partial least squares – age, sex, toric lens, mean K, ICL size, ICL power, CCT, AQD, ACV, LT, ACA distance, pupil diameter, WTW	0.664	< 0.001	96.9	6801.3
	LASSO – ICL size, AQD, ACA distance, LT	0.607	< 0.001	101.8	5769.9
Validation set (N = 196)	Selection from STAAR nomogram - ICL size, WTW, ACD	0.505	< 0.001	112.3	1917.2
	Selection from KS formula – ICL size, ATA (= ACA distance)	0.473	< 0.001	116.9	1920.6
	Forward stepwise selection – age, mean K, ICL size, ICL power, CCT, AQD, ACA distance, LT	0.580	< 0.001	104.9	1917.5
	Backward stepwise selection – age, toric lens, SE, mean K, ICL size, AQD, ACA distance, LT, WTW	0.578	< 0.001	105.7	1927.6
	Partial least squares – age, sex, toric lens, mean K, ICL size, ICL power, CCT, AQD, ACV, LT, ACA distance, pupil diameter, WTW	0.577	< 0.001	105.8	1935.1
	LASSO – ICL size, AQD, ACA distance, LT	0.582	< 0.001	104.7	1894.9

*ACA* anterior chamber angle, *ACV* anterior chamber volume, *AQD* aqueous depth, *ATA* angle-to-angle distance, *CCT* central corneal thickness, *LASSO* least absolute shrinkage and selection operator, *LT* lens thickness, *MAE* mean squared error, *SE* spherical equivalent, *WTW* white-to-white distance

Finally, we selected the LASSO model as the best prediction model for optimal ICL sizing, because it showed the lowest BIC value in both the development (5769.9) and validation (1894.9) sets. The smallest number of predictors in the LASSO model contributed to the best effectiveness for vault prediction. The predicted postoperative vault was calculated according to the LASSO model, as follows:

$$\begin{array}{l}{\rm{Postoperative\ ICL\ vault (}}\mu m)\\= {\rm{ - 1052}}{\rm{.26 + 129}}{\rm{.94}} \times {\rm{AQD (mm) - 134}}{\rm{.54}} \times \\{\rm{ACA\ distance(mm) - 217}}{\rm{.53}} \times {\rm{LT(mm) + }}\\{\rm{283}}{\rm{.62}} \times {\rm{ICL size (mm)}}\end{array}$$

As we assumed that the ideal ICL vault was 500 μm, the optimal ICL size was calculated as follows:

$$\begin{array}{l}{\rm{Optimal\ ICL\ size (mm) = 5}}{\rm{.472 - 0}}{\rm{.458}} \times {\rm{AQD(mm) + }}\\{\rm{0}}{\rm{.474}} \times {\rm{ACA\ distance (mm) + 0}}{\rm{.767}} \times {\rm{LT (mm)}}\end{array}$$

The surgeon selected the ICL lens size from among the four commercial sizes (12.1, 12.6, 13.2, and 13.7 mm) closest to the calculated optimal ICL size. It should be noted that the use of a large lens with a size of 13.7 mm was extremely rare in the development dataset for these equations. We developed a simple web-based calculator application using a final equation (https://soo9028.github.io/iol-prediction-webpage/).

**Fig. 3:**
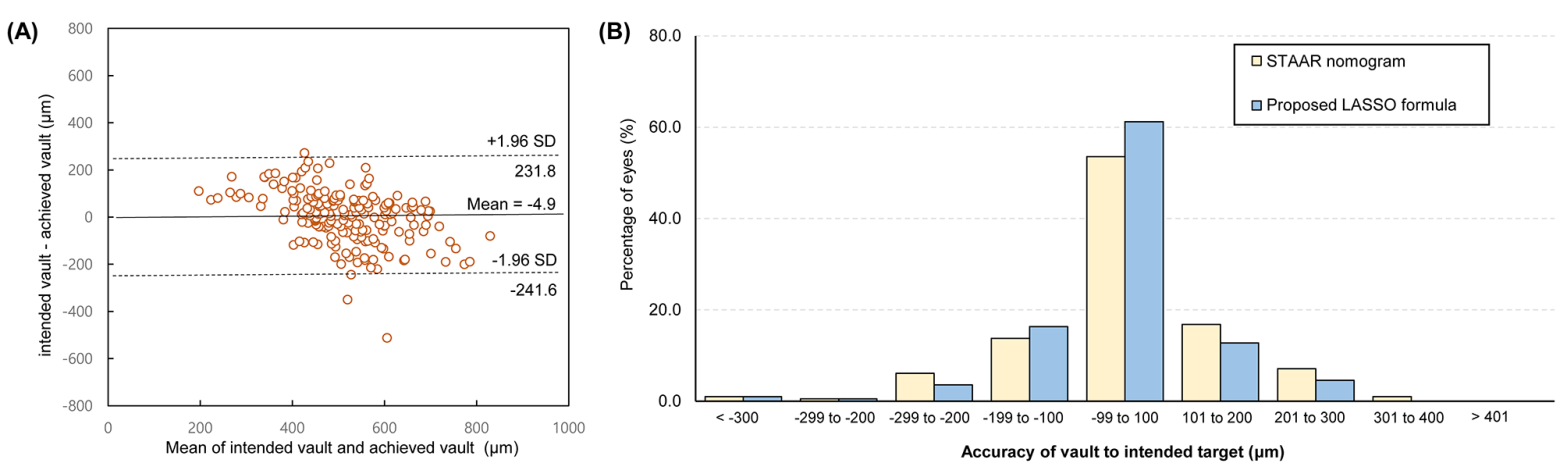
Distribution of the achieved vault against the predicted vault using the LASSO formula in the validation dataset. (A) Bland-Altman plot. (B) Vault errors between the proposed LASSO formula and the STAAR nomogram

Figure 3 shows the validation results for the LASSO model. In the Bland-Altman analysis, the average difference between the real achieved and LASSO-predicted vault values was 4.9 μm, and its standard deviation was 120.8 μm. When we analyzed the distribution of the accuracies according to the predicted vault, the LASSO model showed significantly better prediction performance than the STAAR nomogram-based model (P < 0.001 in the χ^2^ test).

**Fig. 4:**
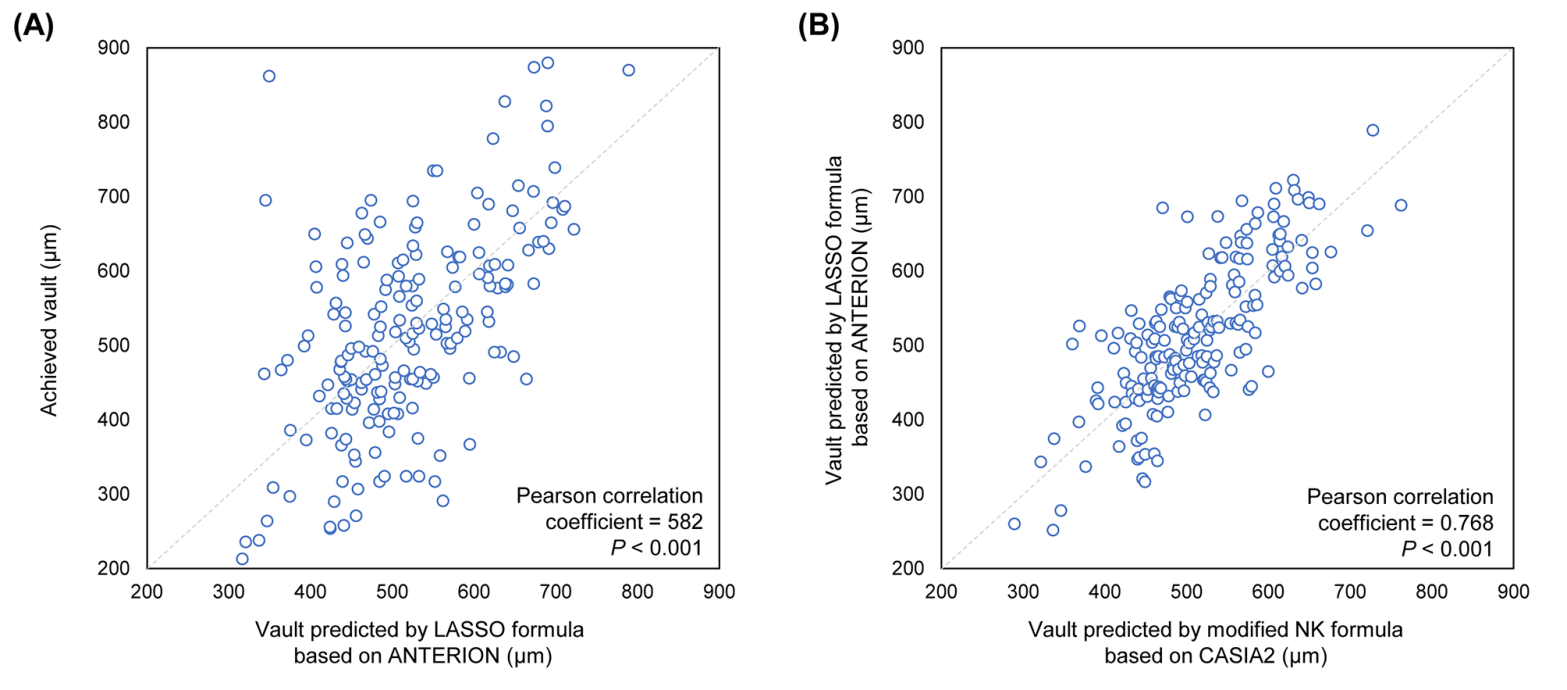
Scatter plots showing the postoperative ICL vault prediction results in the validation dataset. (A) Distribution of the achieved vault against the LASSO predicted vault. (B) Distribution of the LASSO formula against the modified NK formula

**Fig. 5:**
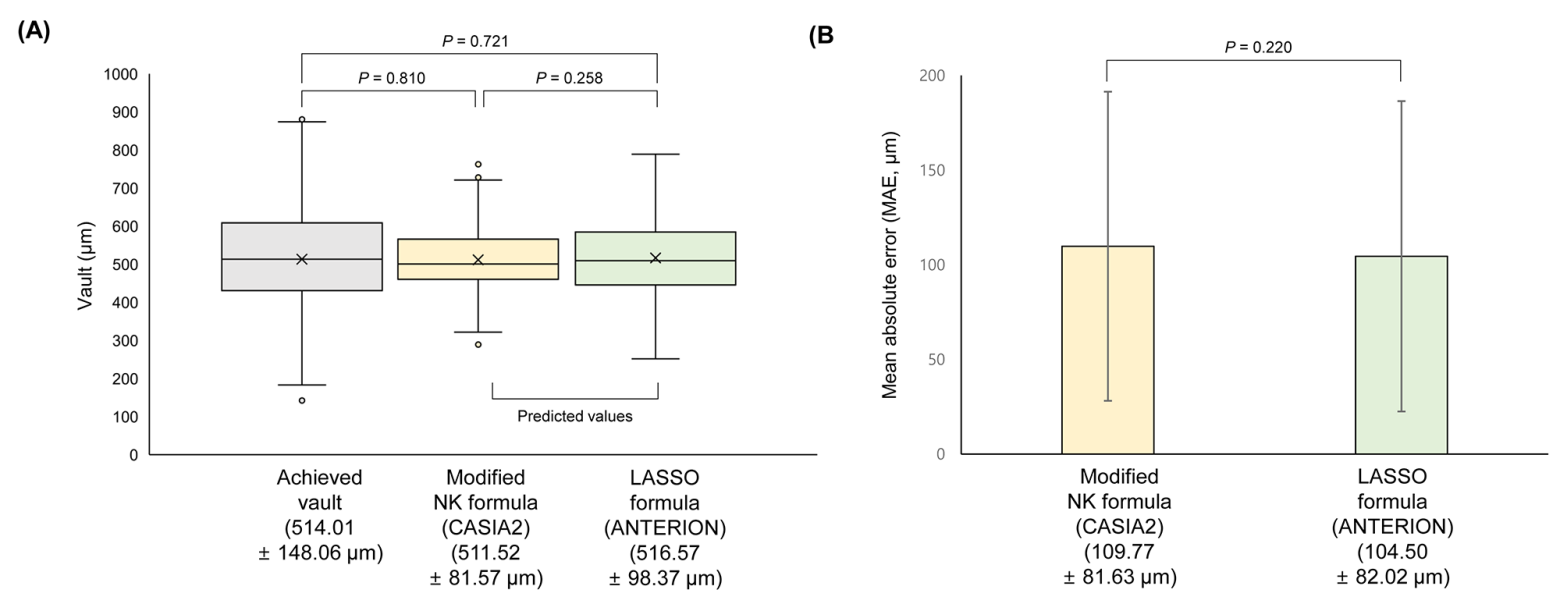
Comparison between the LASSO formula based on ANTERION and the modified NK formula based on CASIA2. (A) Comparison between the achieved vault and prediction results. (B) Mean absolute error (MAE) comparison between the LASSO and modified NK formulas

The developed model was compared with the achieved results and the NK formula in the validation data (Fig. 4). The correlation coefficient between the achieved vaults and the developed LASSO was 0.582 (P < 0.001). The correlation coefficient between the LASSO and NK formulas was 0.768 (P < 0.001). Figure 5 shows that the distributions of the achieved vault (514.01 ± 148.06), NK formula vault prediction (511.52 ± 81.57), and developed LASSO formula vault prediction (516.57 ± 98.37) values showed no significant difference between each other. In addition, the MAEs showed no difference between the two formulas (P = 0.220).

## Discussion

Appropriate IOL sizing with accurate postoperative vault prediction is necessary to achieve safer ICL surgeries and better clinical outcomes. We introduced the first formula to estimate the postoperative ICL vault for the novel AS-OCT device, ANTERION, which has not been used for ICL surgery. Compared to previous studies that focused on regression and feature extraction, our study contributes to the adoption of LASSO and BIC to select a better prediction model for external validation. In addition, we developed a web-based calculator (https://soo9028.github.io/iol-prediction-webpage/) for better accessibility to the developed formula. Our method was almost equivalent to the NK formula based on CASIA2, which has been widely used with reliable performance in predicting postoperative ICL results. As ANTERIORN does not provide CLR values (Fig. 1), the LASSO algorithm selected LT as a significant variable to estimate the impact of the crystalline lens on IOL position. The final formula showed better results than the STAAR nomogram, which used limited predictors, including WTW and ACD. ANTERION provided more significant metrics for estimating the posterior chamber space for ICL implantation than WTW measured at the ocular surface.

Table 4 presents a literature review of ICL size and postoperative vault predictions. Several regression approaches have been developed to achieve vault prediction and optimal ICL sizing. Some of these approaches use UBM to measure the anterior segment [11], whereas others are based on AS-OCT measurements, mainly CASIA2 [15]. UBM (or ultrasound scanner) is a non-invasive technique that uses high-frequency ultrasound transducers (50–100 MHz) for imaging of the anterior segment including iris, crystalline lens, and ciliary body [25]. In a study using UBM, the regression model included ACD, sulcus-to-sulcus [26], and sulcus-to-sulcus lens rise [11]. Recently, a digital ultrasound scanner (ArcScan Inc., Morrison, Colorado) measured the inner diameter of the ciliary body and predicted the postoperative vault more accurately than the UBM [16]. The CASIA2 provides reproducible measurements of the cornea, ACA, iris, and CLR. It has been used to diagnose angle-closure [27] and measure corneal and anterior chamber structures [28]. In a study using CASIA2, the NK formula included ACW and CLR to predict vault size [29]. The ATA from CASIA2 was used as a reliable variable in the KS formula [30]. Recently, the ATA-based formula was revised for more accurate vault prediction at IOL sizes of 12.1, 13.2, and 13.7 mm in a validation study [31]. Most studies have used classic linear regression techniques [29], and PLS has been adopted to achieve more accurate predictions [20]. Recent studies have employed machine-learning approaches using large datasets and many input variables [18]. Although large machine-learning-based models have been applied, too many variables are required to obtain a modest performance gain [32]. A previous study emphasized the ethnic differences and developed a LASSO-based model using MS-39 (Costruzione Strumenti Oftalmici, Florence, Italy) for Caucasian eyes [33]. In this study, we predicted postoperative ICL vault using LASSO regression based on the measurement data from ANTERION. Our model included only three variables (AQD, ACA distance, and LT) and showed the best prediction performance and effectiveness.

**Table 4:** Summary of ICL sizing and postoperative vault prediction using anterior segment imaging domains

Study	Number of study participants	Country	Anterior segment imaging device	Algorithm	Variables for ICL sizing and vault prediction
Manufacturer (STAAR nomogram)	-	-	-	-	WTW, ACD
Dougherty, et al. (2007) [26]	73 eyes of 48 patients	USA	UBM (VuMax-II)	Linear regression	Sulcus-to-sulcus, ICL power
Kojima, et al. (2012) [11]	47 eyes of 25 patients	Japan	UBM (VuMax-II)	Linear regression	ACD, Sulcus-to-sulcus, Sulcus-to-sulcus lens rise
Lee, et al. (2012) [10]	129 eyes of 75 patients	Korea	UBM (Carl Zeiss model 835)	Pearson’s correlation	Sulcus-to-sulcus
Igarashi, et al. (2019) [30] (KS formula v1)	44 eyes of 23 patients	Japan	AS-OCT (CASIA2)	Spearman rank correlation	ATA (= ACA distance)
Nakamura, et al. (2018) [29] (NK formula v1)	46 eyes of 23 patients	Japan	AS-OCT (CASIA2)	Linear regression	ACW, CLR
Nakamura, et al. (2020) [15] (NK formula v2)	81 eyes of 35 patients	Japan	AS-OCT (CASIA2)	Linear regression (stepwise variable selection)	ACW, CLR
Oleszko, et al. (2020) [20]	81 eyes of 43 patients	Poland	Pentacam and AS-OCT (Visante OCT)	Partial least square regression	SE, ATA (= ACA distance), ACD, LE (Visante OCT), axial length, Keratometry (AS-OCT), Corneal radius (Pentacam), ACV
Igarashi, et al. (2021) [31] (KS formula v2)	121 eyes of 65 patients	Japan	AS-OCT (CASIA2)	Corrected KS formula for each ICL size (discrete function)	ATA (= ACA distance), vault prediction by KS formula × 0.8 at a size of 12.1 mm and KS formula × 1.3 at sizes of 13.2 and 13.7 mm
Kamiya, et al. (2021) [18]	1745 eyes of 1745 patients	Japan & South Korea	AS-OCT (CASIA2)	Random forest	Age, sex, refractive power (sphere and cylinder), SE, BCVA, toric lens, WTW, ACD, ATA (= ACA distance), CLR, ACW, LV, CCT, AOD500, TIA500
Kang, et al. (2021) [5]	3506 eyes of 1753 patients	South Korea	AS-OCT (CASIA2)	XGBoost + LightGBM	age, sex, SE, ACD, ACW, ATA, WTW, CLR, pupil size, CCT, toric lens, ICL power
Shen, et al. (2021) [32]	6297 eyes of 3536 patients	China	Pentacam, IOL-Master and UBM (Quantel)	Random forest	ACA, pupil size, axial length, Keratometry, refractive power (sphere and cylinder), ACD, CCT, WTW, SE, toric lens, ICL power, time after surgery
Reinstein, et al. (2022) [16]	147 eyes	United Kingdom	Ultrasound scanner (ArcScan)	Linear regression	ciliary body inner diameter, Sulcus-to-sulcus lens rise, pupil diameter
Rocamora, et al. (2022) [33]	115 eyes of 59 patients	Belgium	AS-OCT (MS-39)	LASSO	Keratometry, Corneal volume, CLR, pupil size, iris diameter, ICL power
This study	894 eyes of 471 patients	South Korea	AS-OCT (ANTERION)	LASSO	AQD, ACA distance, LT

*ACA* anterior chamber depth, *ACD* anterior chamber angle, *ACD* anterior chamber depth, *ACV* anterior chamber volume, *ACW* anterior chamber width, *AOD500* average nasal and temporal angle open distance at 500 μm, *AQD* aqueous depth, *AS-OCT* anterior segment optical coherence tomography, *ATA* angle-to-angle distance, *CCT* central corneal thickness, *CLR* crystalline lens rise, *LASSO* least absolute shrinkage and selection operator, *LE* lens elevation, *LT* lens thickness, *LV* lens vault, *SE* spherical equivalent, *TIA500* average nasal and temporal trabecular iris angle at 500 μm, *UBM* ultrasound biomicroscopy, *WTW* white-to-white distance

We developed a new ICL sizing formula based on ANTERION. Using our formula, ANTERION can be used for optimal ICL sizing in the clinic. Because the measurements from two swept-source AS-OCT devices, ANTERION and CASIA2, are not interchangeable [13], the direct use of the anatomic feature values from ANTERION in the formulas for CASIA2 is limited and does not guarantee accurate prediction. According to the literature, ANTERION has several advantages over other AS-OCT devices [13]. It can visualize the ciliary muscle and entire crystalline LT with a high resolution of < 10 μm and can also measure the axial length of the eye. The posterior lens surface cannot be visualized using other AS-OCT techniques, such as CASIA2 and RTVue (Optovue Inc., Fremont, California, USA). ANTERION uses the image averaging technique, which is a new feature of AS-OCT devices, to improve the signal-to-noise ratio to measure wider and deeper structures of the anterior segments [34]. It also showed high repeatability for anatomical measurements of the anterior segments. As expected, the axial measurements (AQD and LT) and width (ACA distance) were selected for the final LASSO regression model, similar to the NK formula using CASIA2 [15]. The difference is that our formula contains ACD and LT without using a crystalline lens rise or crystalline lens vault (LV). In this study, we used only measurements captured using a fully automated process. We did not collect LV measurements because they vary depending on the light conditions and study population [5],[35]. According to our results, LT is also highly correlated with postoperative ICL vault, and the combination of LT and other measurements showed better predictive performance than the STAAR nomogram-based and KS formula-based methods, which do not contain LT information.

The simplest linear model, LASSO, outperformed the complex regression approaches, which usually achieved good results in other studies. This may be due to the occurrence of overfitting to the development set in complex regression models using many variables. This result indicates that none of the predictors improved the performance in predicting postoperative ICL vaults. Previous studies have also shown that LASSO with fewer predictors outperforms other complex regressions [22],[23]. Because LASSO assigns zero to most predictors with low impact, it is considered a good feature selection technique [19]. In our study, because the predictors from ANTERION had many intercorrelated associations and different variations, the greater capacity of other complex regressions with more variables seems to lead to overfitting. The standard regression provided insightful results regarding the impact of each variable on vault calculation (Table 2). However, standard linear regression assumes that all predictors are independent of each other [36]. Therefore, it has a multicollinearity problem and works worse than the LASSO model. LASSO can control the multicollinearity between predictors by reducing predictors, and can finally avoid an overfitting problem [37]. The BIC metric successfully evaluated both the regression performance and the effectiveness of ICL vault prediction by penalizing the number of predictors. BIC found that the LASSO-based formula is the most robust model for predicting the ICL vault because it avoids overfitting.

This study had several limitations. First, it was conducted in a single center involving the East Asian population. There may be differences in the anatomical features of the anterior segment between different ethnic groups [38]. In addition, there was no external validation to evaluate the performance of the ICL sizing. Therefore, surgeons in other centers should carefully apply the developed formula, particularly in different ethnic populations. Second, the use of a large lens with a size of 13.7 mm was extremely rare in our study. Therefore, we could not confirm whether our formula can be used for the largest ICL size. Third, we did not consider other ocular measurements such as axial length or corneal parameters. ANTERION provides axial length measurements, but we did not include them to focus on the anterior chamber structure. Anterior chamber shapes have also been associated with factors measured by other modalities in previous studies [39],[40]. Fourth, we did not consider ICL power in this study. According to a previous study, postoperative refraction was associated with the postoperative IOL vault level [41]. This means that our formula can be integrated into the calculation of the ICL refractive power.

## Conclusion

To our knowledge, this is the first study to develop a postoperative ICL vault prediction model based on the ANTERION AS-OCT. It can be used to optimize the ICL size to avoid postoperative complications. In the present study, the LASSO regression model using AQD, ACA distance, and LT showed better performance in estimating ICL vault and ICL sizing than other regression models. Because our model was developed based on the East Asian population, further studies are needed to explore the role of this prediction model in different populations.

## Electronic supplementary material

Below is the link to the electronic supplementary material.

Supplementary Material 1

## Data Availability

The datasets generated and/or analyzed during the current study are not publicly available due to personal privacy concerns but are available from the corresponding author on reasonable request. The developed calculator is available at https://soo9028.github.io/iol-prediction-webpage/.

## Abbreviations

ACA: Anterior chamber angle
ACD: Anterior chamber depth
ACV: Anterior chamber volume
ACW: Anterior chamber width
AQD: Aqueous depth
AS-OCT: Anterior segment optical coherence tomography
ATA: Angle-to-angle distance
BIC: Bayesian information criterion
CCT: Central corneal thickness
CLR: Crystalline lens rise
ICL: Intraocular collamer lens
IOL: Intraocular lens
LASSO: Least absolute shrinkage and selection operator
LT: Lens thickness
LV: Lens vault
MAE: Mean squared error
PLS: Partial least squares
STS: Spur-to-spur
UBM: Ultrasound biomicroscopy
WTW: White-to-white distance
