# Supplementary material for: Development of an implantable collamer lens sizing model: a retrospective study using ANTERION swept-source optical coherence tomography and a literature review
Source: BMC Ophthalmol. 2023 Feb 10;23:59. doi: 10.1186/s12886-023-02814-7 (PMC9921691; doi:10.1186/s12886-023-02814-7)
Supplement: Supplementary file 1 — Supplementary Material 1 [file 12886_2023_2814_MOESM1_ESM.docx]

**Summary Box**

**What was known before:**

Anterior segment optical coherence tomography has recently been developed to more accurately measure the anatomy of the anterior segment of the eye for ICL surgery. However, no studies have been conducted on lens sizing using biometry from ANTERION.

**What this study adds:**

We developed a postoperative ICL vault prediction model based on ANTERION AS-OCT to optimize the ICL size to avoid postoperative complications.
